# Magnetic resonance phase contrast imaging in children with pulmonary artery hypertension

**DOI:** 10.1186/1532-429X-11-S1-P246

**Published:** 2009-01-28

**Authors:** Brian Fonseca, Dunbar Ivy, Craig Lanning

**Affiliations:** 1 The Children's Hospital, Denver, Denver, CO USA; 2 University of Colorado, Denver, CO USA

**Keywords:** Stroke Volume, Pulmonary Artery Pressure, Acceleration Time, Main Pulmonary Artery, Flow Index

## Objective

To compare the flow indices in the main pulmonary artery (MPA) of children with pulmonary artery hypertension (PAH) to children without PAH, non-invasively, with magnetic resonance phase contrast imaging.

## Background

MPA flow indices obtained by magnetic resonance phase contrast imaging have recently been used to diagnose PAH in adults. To our knowledge these techniques have not been previously used in children.

## Methods

Over a four year period, pediatric patients with PAH (mean PA pressure > 25 mmHg on right heart catheterization) who had undergone cardiac MRI with phase contrast imaging of the MPA were included in the study (6 patients). Seven control patients who underwent cardiac MRI at our institution for reasons other than PAH were also included. There was no significant difference in age, weight or sex between the two groups.

The phase contrast images were analyzed by an observer blinded to the mean pulmonary artery pressure for: minimum and maximum MPA area; MPA area strain; maximum and mean MPA velocity; minimum, maximum, and mean flow; stroke volume; acceleration time (AT) [time from the onset of systolic flow to peak systolic flow]; ejection time (ET) [time from onset of systolic flow to the time of minimum flow]; and acceleration time to ejection time ratio (AT/ET).

Differences between the PAH and the control groups were calculated with an unpaired, two-tailed student T test assuming equal variance.

## Results

There were significant differences between the PAH and control group for minimum MPA area (779 mm^2^ ± 190 vs. 500 mm^2^ ± 128, p = 0.009), MPA area strain (22% ± 12 vs. 55.5% ± 17, p = 0.002), mean velocity (7 cm/s ± 1.6 vs. 11.9 cm/s ± 3.6, p = 0.01), and ejection time (295 ms ± 56 vs. 559 ms ± 239, p = 0.02) There was a trend towards significance in MPA stroke volume (43 ml ± 14 vs. 64 ml ± 21, p = 0.06) and acceleration time (111 ms ± 16 vs. 146 ms ± 37, p = 0.06). No significant difference was found in MPA maximum area (945 mm2 ± 226 vs. 766 mm2 ± 177, p = 0.13), minimum MPA flow (-40 ml/s ± 20 vs. -29 ml/s ± 24, p = 0.40), maximum MPA flow (298 ml/s ± 63 vs. 320 ml/s ± 97, p = 0.64), mean flow (61 ml/s ± 15 vs. 82 ml/s vs. ± 25, p = 0.1), maximum velocity (96 cm/s ± 20 vs. 101 cm/s ± 35, p = 0.76) or AT/ET ratio (0.39 ± 0.1 vs. 0.30 ± 0.12, p = 0.17).

## Discussion

Our findings largely agreed with those found in adult patients with PAH. While only minimum PA diameter achieved significance, both minimum and maximum PA area were larger in the PAH group. Taken in conjunction with the significantly lower MPA strain found in the PAH group, these findings suggest lower compliance in the main MPA in children with PAH.

Although maximum MPA velocities were not significantly different between the two groups, mean MPA velocity was significantly lower in the PAH group. No significant difference was found in minimum, maximum or mean MPA flow between the two groups.

The PAH group showed a trend towards lower acceleration times, a finding that has been demonstrated in adults. Unlike adults with PAH, the children in this study had significantly lower than normal ejection times.

## Conclusion

Magnetic resonance phase contrast imaging reveals significant derangements in MPA flow dynamics in children with PAH which may distinguish them from children with normal pulmonary artery pressures. In contrast to findings in adults, children with PAH have significantly shorter ejection times than normal, a novel finding.

